# Sevoflurane Induces Exaggerated and Persistent Cognitive Decline in a Type II Diabetic Rat Model by Aggregating Hippocampal Inflammation

**DOI:** 10.3389/fphar.2017.00886

**Published:** 2017-11-29

**Authors:** Dongliang Li, Lingling Liu, Liang Li, Xingang Li, Bin Huang, Changqing Zhou, Zhaohang Zhang, Chunling Wang, Ping Dong, Xiyan Zhang, Bo Yang, Li Zhang

**Affiliations:** ^1^Department of Anesthesiology, Qilu Hospital, Shandong University, Jinan, China; ^2^Jining Health School of Shandong Province, Jining, China; ^3^Department of Neurosurgery, Qilu Hospital and Brain Science Research Institute, Shandong University, Jinan, China

**Keywords:** sevoflurane, type 2 diabetes mellitus, cognitive decline, hippocampal inflammation, microglia

## Abstract

Recent studies show that a moderate duration of sevoflurane, one of the most commonly used volatile anesthetics in clinical practice, does not induce cognitive impairment in animals under physiological conditions. However, the influence of sevoflurane on cognitive function under diabetic conditions remains unclear. The aim of this study was to determine whether sevoflurane causes cognitive decline in a rat model of type 2 diabetes mellitus (DM) and if so, to explore a possible underlying mechanism. Diabetic Goto–Kakizaki (GK) rats and non-diabetic Wistar rats underwent 2.6% sevoflurane for 4 h or sham (control) exposure. Cognitive function and hippocampal inflammation were assessed 1 week and 5 months after sevoflurane or sham exposure. Compared with Wistar control rats, GK control rats exhibited shorter freezing times in Trace fear conditioning task 1 week after exposure, took longer to locate the submerged platform and had shorter dwell-time in the target quadrant in Morris Water Maze task 5 months after exposure. GK rats that received sevoflurane not only exhibited less freezing times 1 week after exposure, but also spent more time to locate the submerged platform and had less dwell-time in the target quadrant, compared with GK control rats. Molecular studies revealed that the levels of pro-inflammatory cytokines and activated microglia in the hippocampus were higher in GK control rats than those in Wistar control rats at both time points and were further increased in GK rats receiving sevoflurane. Wistar rats that received sevoflurane and Wistar control rats did not differ in any cognitive performance and molecular assessment. The results suggest that diabetic GK rats exhibit cognitive dysfunction probably due to increased hippocampal inflammation, and that sevoflurane induces exaggerated and persistent cognitive decline in GK rat by aggregating hippocampal inflammation.

## Introduction

Post-operative cognitive dysfunction (POCD) is generally defined as a decline in cognitive function that occurs in patients after anesthesia and surgery when compared to their preoperative cognitive status. POCD occurs in approximately 10% of all surgical patients and 40% of elderly patients at age 65 and older (Moller et al., 1998), and is associated with increased mortality, decreased quality of life and increased dependency (Steinmetz et al., 2009). Although the cause of POCD remains to be determined, general anesthesia alone is now being recognized as a potentially significant risk to cognitive performance at both extremes of age. A large body of experimental studies as well as some retrospective clinical evidence have showed that exposure to anesthetics could be detrimental to cognitive development in young subjects, and might also contribute to cognitive impairment in the elderly (Jevtovic-Todorovic et al., 2013; Shoair et al., 2015). Of note, anesthesia-induced cognitive impairment may depend on age, anesthetic agent, dosage, duration, and number of exposures (Hudson and Hemmings, 2011; Shen et al., 2013; Callaway et al., 2015). Additionally, the presence of chronic diseases may also increase the likelihood of developing cognitive impairment or cause exacerbation of pre-existing cognitive decline after anesthesia (Feng et al., 2013; Yang et al., 2014; Yue et al., 2015).

Diabetes mellitus (DM) is an increasingly common medical condition affecting approximately 347 million people worldwide, and about 90% of them have type 2 DM (Nyenwe et al., 2011). An estimated 25% of diabetic patients will require surgery (Nyenwe et al., 2011). Recent evidence has suggested that DM is associated with cognitive dysfunction and is an important risk factor for dementia (Jiang et al., 2012; Kariharan et al., 2015; Liu et al., 2016; Zuloaga et al., 2016).

Sevoflurane is one of the most commonly used volatile anesthetic agents for the maintenance of anesthesia in surgical patients, including patients with type 2 DM (Bulte et al., 2014). Sevoflurane has favorable clinical characteristics such as rapid pharmacokinetics and lack of airway irritability. Previous studies have demonstrated that a moderate duration of sevoflurane (2–3% for 2 or 4 h) does not induce cognitive impairment in both adult and aged animals (Callaway et al., 2012; Shen et al., 2013). These studies, however, have been conducted in animals under physiological conditions and clinical studies of POCD exclude patients who are already cognitively impaired. To date, however, the influence of sevoflurane on cognitive dysfunction under diabetic conditions has not been examined. The present study sought to determine whether sevoflurane might exaggerate cognitive dysfunction in a rat model of type 2 DM and if so, to explore a possible underlying mechanism, focusing on sevoflurane-induced change in neuroinflammation that has been implicated in pathophysiology of cognitive impairment (Moreira et al., 2007; Li et al., 2013b; Zhao et al., 2013; Zuloaga et al., 2016).

## Materials and Methods

### Animals

All experiments and procedures were approved by the Animal Care and Use Committee at Shandong University, and performed in accordance with the guidelines of the Animal Care and Use Committee at Shandong University. All efforts were made to minimize animal suffering and the number of animals used in the study.

Twelve-week-old male diabetic Goto–Kakizaki (GK) rats and age-matched male non-diabetic Wistar rats were purchased from SLAC Laboratory Animal, Co., Ltd. (Shanghai, China). GK rat is a genetic non-obese model of type 2 DM with relatively stable hyperglycemia, early hyperinsulinemia and insulin resistance and later insulin deficiency (Wang et al., 2013). This model is prone to developing cognitive deficits and learning impairments along aging and has been considered to be one of the useful animal models for studying the pathogenesis of type 2 DM and its neurological complications (Moreira et al., 2007; Li et al., 2013b; Hussain et al., 2014). A previous study has shown that GK rats display cognitive dysfunction at 7–8 months of age, we therefore used 9-month-old GK rats to examine the influence of sevoflurane on cognitive dysfunction under diabetic conditions in our study. All Animals were maintained in environmentally controlled rooms and were granted free access to standard rat chow and tap water.

### Experimental Design

At 9 months of age, hyperglycemia was confirmed via tail vein blood samples, the animals were then assigned to the following experimental groups (*n* = 20 rats per group): (1) Wistar control rats (Wistar + CON), (2) Wistar rats treated with sevoflurane (Wistar + SEV), (3) GK control rats (GK + CON), and (4) GK rats treated with sevoflurane (GK + SEV).

Sevoflurane exposure was performed as previously described (Yue et al., 2015). Briefly, rats were placed in a temperature controlled chamber with two interfaces that were connected to an anesthesia machine and a multi-gas monitor, respectively. For rats assigned to sevoflurane exposure groups, 2.6% sevoflurane was provided by a humidified 30% O_2_ carrier gas from a calibrated vaporizer for 4 h. Rats assigned to control groups were also placed in the same chamber except no sevoflurane was provided (sham exposure). The concentrations of sevoflurane, O_2_ and CO_2_ in the chamber were continuously monitored by a gas analyzer (Datex Ohmeda, Mississauga, ON, Canada). The rectal temperature was maintained at 37°C during the experiment. Blood pressure (BP) and heart rate were measured hourly with a CODA Monitor (Kent Scientific, Corp., Torrington, CT, United States). Arterial blood gasses were measured in some rats (*n* = 6 for each group) before and immediately after sevoflurane exposure.

Ten rats from each group were used for open field test and trace-fear conditioning 7 days after sevoflurane exposure to determine the short-term effect of sevoflurane on cognitive function. The rest of animals from each group (*n* = 10 rats per group) were used for Morris water maze (MWM) 5 months after sevoflurane exposure (at the age of 14 months) to determine long-term effect of sevoflurane on cognitive function. Immediately after trace-fear conditioning or MWM assessments, animals were sacrificed to collect brains and blood for molecular and biochemical analyses or perfused transcardially with 4% paraformaldehyde for immunofluorescent study.

### Open-Field Test

General locomotor activity was assessed by open-field test as previously described (Feng et al., 2013). Each rat was placed in an open field apparatus. The floor of the apparatus was subdivided into 25 blocks (9″ square) with thin white stripes, and animal activity was recorded via a digital camera. The number of line crossings and rearings performed in 5 min time period was scored. The apparatus was cleaned with 75% alcohol and rinsed with water to avoid the presence of olfactory cues following each test.

### Trace Fear Conditioning

Hippocampal-dependent learning and memory tasks were assessed by trace fear conditioning (TFC) as previously described (Konopka et al., 2010; Gambus et al., 2015) with minor modifications. TFC training and testing were carried out in a room with overhead fluorescent light and a ventilation fan providing background noise (65 db). During the training, rats were placed in the experimental chamber (Med Associates, Inc.) and allowed to adapt to this context for 3 min, after which they received three consecutive pairs of tone (80 dB, 5 kHz, 20 s) and foot shock (0.8 mAmp, 2 s) with an empty trace interval of 20 s and a break between each pair of 3 min.

Sevoflurane exposure was performed within 30 min after training. Memory of the learned fear was assessed 7 days later by returning the animals into the original chamber without tone and shock. Behavior for each animal was recorded and scored every 5 s during the 5 min observation period. A percentage was calculated using the formula 100 × f/n, where f is the number of freezing events (absence of all movement except for respiration) per animal and n is the total number of observations per animal.

### Morris Water Maze

Five months after sevoflurane exposure, spatial and related forms of learning and memory were assessed by MWM, as previously described (Zhang et al., 2016). Briefly, a 10-cm diameter platform was placed 1 cm above the water surface in a circular tank (diameter, 150 cm; depth, 50 cm). A flag was placed on the platform to increase its visibility. Rat was gently released in the water and emerged from the water onto the platform within 120 s. If the rat failed to find the platform, place the rat on the raised platform and allow it to stay there for 20 s before being removed from the pool. A different platform location was used for each subsequent trial. This cueing procedure enables the rats to acknowledge that they can escape the water by finding a platform.

After cueing procedure, the platform in the third quadrant was submerged 1.5 cm below the water level and the place trials were carried out for 4 days to examine the rats’ ability to obtain spatial information. A black curtain was used to surround the pool to eliminate confounding visual cues. Each rat received four trials per day in each of the four quadrants of the swimming pool. On each trial, rats were placed into the water facing the pool wall, and allowed to search for the platform for a maximum of 120 s and stay on the platform for 20 s before being removed from the pool. A rat that failed to find the platform within 120 s was guided to the platform and stayed there for 20 s. For all training trials, the time required for a rat to reach the hidden platform (escape latency), swimming speed and swimming path were automatically digitized and recorded. The average of four trials performed each day for each animal was calculated as the escape latency.

The day after the completion of the hidden platform training, probe trials were conducted to examine whether the rats had developed a spatial bias for the former platform quadrant. The platform in the third quadrant was removed from the pool and rats were allowed to swim for 120 s in any of the four quadrants of the swimming pool. The number of crossings over the original position of the platform and the percent time spent in each quadrant were recorded.

### Western Blot Analysis

Protein levels of pro-inflammatory cytokines in the hippocampus were analyzed by western blot as previously described (Zhang et al., 2016). Briefly, the hippocampus was quickly dissociated from brain and homogenized in an ice-cold lysis buffer with protease inhibitor. The protein concentration was determined by a Bradford assay using BSA as the standard. Samples were electrophoresed on 12% sodium dodecyl sulfate (SDS)-polyacrylamide gels, and the gels were transferred to polyvinylidene difluoride (PVDF) membranes. After blocking for 1 h in 5% non-fat dry milk, the membranes were incubated with primary antibodies to tumor necrosis factor (TNF)-α, interleukin (IL)-1β, IL-6, and β-actin (Santa Cruz Biotechnology, Inc., Santa Cruz, CA, United States) at 4°C overnight. Membranes were then washed and incubated at room temperature with HRP-conjugated second antibody (Santa Cruz Biotechnology, Inc.) for 1 h. The enhanced chemiluminescence detection system (Amersham) was applied to visualize the immunoreactive bands, and the band densities were analyzed with ImageJ software (National Institutes of Health). All data were normalized by β-actin.

### Immunofluorescent Study

Rats were perfused transcardially with heparinized saline followed by 4% paraformaldehyde. Brains were removed and post-fixed overnight in 4% paraformaldehyde at 4°C and then immersed in 30% sucrose. Serial coronal sections of 20-μm thickness were cut using a cryostat. Immunofluorescent staining was performed as previously described with modifications (Rana et al., 2010; Li et al., 2013a). Briefly, brain sections were incubated by 0.5% H_2_O_2_ for 30 min followed by 10% normal horse serum for 60 min to facilitate antibody penetration. After washing three times, the sections were incubated with anti-rat CD11b primary antibody (clone OX-42) (1:100, Chemicon, Temecula, CA, United States) at 4°C overnight. Subsequently, the sections were incubated for 2 h in an anti-mouse secondary antibody (Alex Fluor 488, 1:200; Invitrogen, Carlsbad, CA, United States) followed by washing three times. Immunofluorescent staining for microglia was visualized with a confocal microscope (Zeiss LSM 510; Carl Zeiss, Thornwood, NY, United States). The number of activated microglia, defined by stronger immunofluorescent staining for the marker CD11b (clone OX-42), the presence of a clearly enlarged soma and marked changes in the appearance of the processes (Rana et al., 2010), were counted in several 0.2 mm × 0.2 mm squares and expressed as a percentage of the total number of microglia.

### Biochemical Assays

Blood samples were collected and placed on ice immediately. Plasma was isolated by centrifugation at 2500 *g* for 15 min at 4°C. Plasma glucose levels were measured using a glucose analyzer (Prestige Smart System). Plasma insulin levels were measured by commercially available rat ELISA kit (Invitrogen, Camarillo, CA, United States).

### Statistical Analysis

Data were analyzed using a two-way ANOVA. Factors were identified as diabetes (Wistar and GK) and sevoflurane exposure (CON and SEV). Tukey’s *post hoc* test was subsequently used for comparisons between groups. In MWM test, the number of original platform (the third quadrant) crossings and the percent time spent in the third quadrant were analyzed using a two-way ANOVA followed by Tukey’s *post hoc* test. Data are presented as mean ± SE. Values were considered statistically significant when *P* < 0.05.

## Results

### Sevoflurane Induces Short-Term Memory Decline in GK Rats But Not in Wistar Rats

**FIGURE 1:**
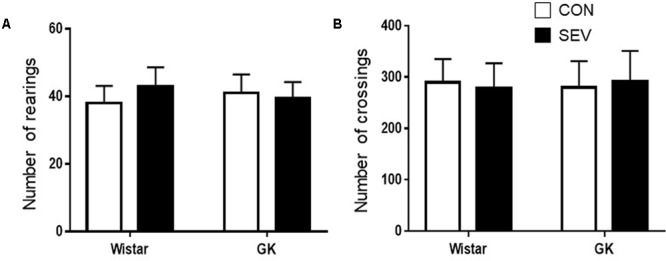
Baseline locomotion assessed by open field test in type 2 diabetic Goto–Kakizaki (GK) rats and non-diabetic Wistar rats at 9 months of age. There were no differences among four experimental groups in the number of rearings **(A)** and the number of crossings **(B)**. Data are presented as mean ± SE (*n* = 10 for each group).

To exclude possible locomotor dysfunction that might confound the cognitive assessment, open-field test was conducted to assess spontaneous activity prior to cognitive test. There were no differences among four experimental groups in the number of rearings (**Figure 1A**) and the number of crossings (**Figure 1B**).

**FIGURE 2:**
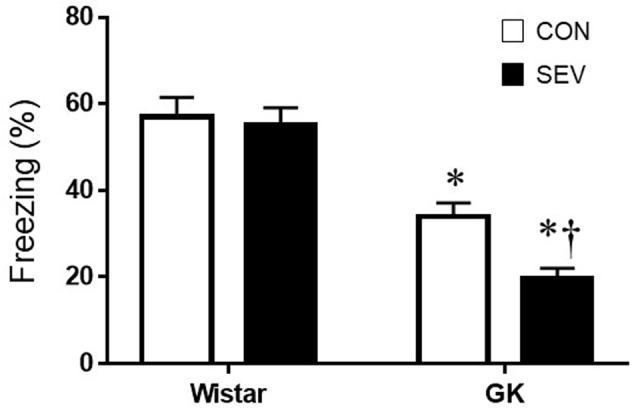
Fear conditioning in type 2 diabetic Goto–Kakizaki (GK) rats and non-diabetic Wistar rats 7 days after sevoflurane (SEV) or sham (control, CON) exposure. Freezing times was shorter in GK + CON rats than Wistar +CON rats and was further reduced in GK + SEV rats. Of note, freezing times was similar between two Wistar groups. Data are presented as mean ± SE (*n* = 10 for each group). ^∗^*P* < 0.05 vs. respective Wistar rats; ^†^*P* < 0.05 vs. GK + CON rats.

Short-term memory was assessed by the percentage of time spent freezing when rats were placed in the same TFC training context 7 days after sevoflurane exposure. Notably, GK control rats exhibited significantly less freezing when compared with Wistar control rats at 9 months of age [*F*(1,36) = 65.05, *P* < 0.01] (**Figure 2**), indicating that type 2 DM causes impairment in hippocampal-dependent memory at this time point. Sevoflurane exposure did not alter freezing in Wistar rats but further reduced freezing in GK rats, compared with respective control groups [*F*(1,36) = 5.13, *P* < 0.05].

### Sevoflurane-Induced Cognitive Decline in GK Rats Is Persistent

**FIGURE 3:**
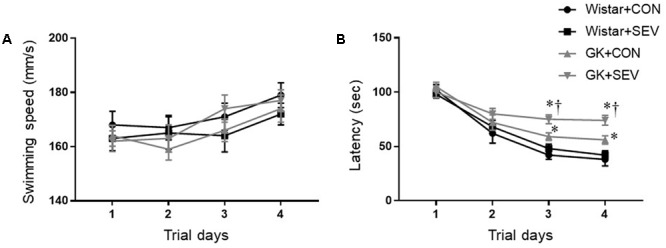
The ability of spatial information acquisition assessed by Place trial in type 2 diabetic Goto–Kakizaki (GK) rats and non-diabetic Wistar rats 5 months after sevoflurane (SEV) or sham (control, CON) exposure. There was no difference across four experimental groups in swimming speed throughout 4 consecutive days **(A)**. However, both groups of GK rats took longer to locate the submerged platform than Wistar rats on the last two trials, and the GK + SEV rats spent more time to find the submerged platform than GK + CON rats **(B)**. Data are presented as mean ± SE (*n* = 10 for each group). ^∗^*P* < 0.05 vs. respective Wistar rats; ^†^*P* < 0.05 vs. GK+CON rats.

Five months after sevoflurane exposure, place trial revealed that there was no difference across four experimental groups in swimming speed throughout 4 consecutive days (**Figure 3A**), suggesting that the motor ability of rats was not affected by type 2 DM or sevoflurane. The escape latency to the submerged platform did not differ between Wistar control rats and Wistar rats that received sevoflurane exposure (**Figure 3B**). In contrast, both groups of GK rats exhibited significantly longer latency to find the submerged platform than the Wistar rats on the last two trials [day 4: *F*(1,36) = 26.88, *P* < 0.01], and the GK rats that received sevoflurane exposure had much higher latency than GK control rats [day 4: *F*(1,36) = 5.204, *P* < 0.05].

**FIGURE 4:**
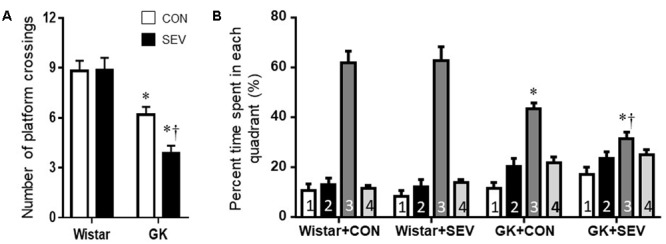
The memory retention capabilities assessed by Probe trial in type 2 diabetic Goto–Kakizaki (GK) rats and non-diabetic Wistar rats 5 months after sevoflurane (SEV) or sham (control, CON) exposure. The number of original platform (the third quadrant) crossings **(A)** and the percent time spent in the third quadrant **(B)** were reduced in both groups of GK rats than Wistar rats. Moreover, the number of platform crossings and the percent time spent in the third quadrant were less in GK + SEV rats than those in GK + CON rats. Data are presented as mean ± SE (*n* = 10 for each group). ^∗^*P* < 0.05 vs. respective Wistar rats; ^†^*P* < 0.05 vs. GK+CON rats.

On the probe trial, no differences between the two Wistar groups were observed for the number of platform crossings (**Figure 4A**) and the percent time spent in third quadrant (**Figure 4B**). Compared with Wistar rats, both groups of GK rats displayed significantly reduced number of platform crossings [*F*(1,36) = 56.31, *P* < 0.01] and percent time spent in third quadrant [*F*(1,36) = 35.31, *P* < 0.01]. Moreover, the reductions in the number of platform crossings [*F*(1,36) = 4.71, *P* < 0.05] and the percent time spent in third quadrant [*F*(1,36) = 4.25, *P* < 0.05] were greater in GK rats receiving sevoflurane exposure than GK control rats.

### Sevoflurane Aggravates Hippocampal Inflammation and Microglia Activation in GK Rats

**FIGURE 5:**
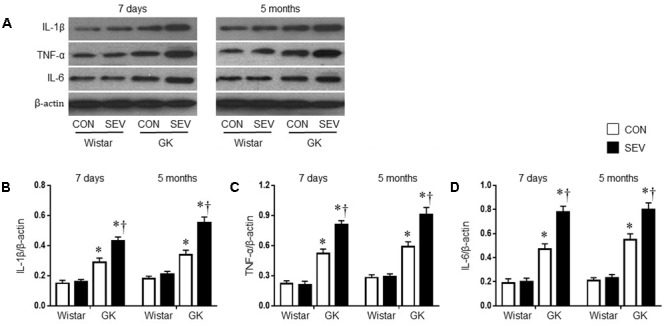
Representative Western blots **(A)** and quantitative comparison of protein levels for pro-inflammatory cytokines IL-1β **(B)**, TNF-α **(C)**, and IL-6 **(D)** in the hippocampus in type 2 diabetic Goto–Kakizaki (GK) rats and non-diabetic Wistar rats 7 days and 5 months after sevoflurane (SEV) or sham (control, CON) exposure. The levels of pro-inflammatory cytokines in the hippocampus were higher in GK + CON rats than those in Wistar + CON rats at both time points and were further increased in GK + SEV rats. Data are presented as mean ± SE (*n* = 6 for each group). ^∗^*P* < 0.05 vs. respective Wistar rats at the same time point; ^†^*P* < 0.05 vs. GK + CON rats at the same time point.

Given the findings that sevoflurane exposure induced cognitive decline in GK rats, we next investigated the possible underlying mechanism. Pro-inflammatory cytokines, particularly IL-1β, TNF-α and IL-6, are associated with cognitive impairment (Perry, 2004; Goshen et al., 2007; Teeling and Perry, 2009; Patanella et al., 2010). We therefore assessed the effects of the sevoflurane exposure on the levels of IL-1β, TNF-α, and IL-6 in the hippocampus, a brain structure that is critical for learning and memory. Western blot analysis revealed that the protein levels of IL-1β, TNF-α, and IL-6 in the hippocampus, measured at 7 days or 5 months after sevoflurane exposure, were not different between the two Wistar groups (**Figure 5**). However, the protein levels of IL-1β, TNF-α, and IL-6 in the hippocampus in both groups of GK rats were increased at 7 days after sevoflurane exposure [IL-1β: *F*(1,20) = 76.65, *P* < 0.01; TNF-α: *F*(1,20) = 144.8, *P* < 0.01; IL-6: *F*(1,20) = 114.6, *P* < 0.01] and remained at high levels 5 months after sevoflurane exposure [IL-1β: *F*(1,20) = 78.49, *P* < 0.01; TNF-α: *F*(1,20) = 96.06, *P* < 0.01; IL-6: *F*(1,20) = 121.7, *P* < 0.01], compared with Wistar rats. Of note, the increases in the protein levels of these pro-inflammatory cytokines were greater in GK rats receiving sevoflurane exposure than GK control rats at both time points [7 days: IL-1β: *F*(1,20) = 10.26, *P* < 0.01; TNF-α: *F*(1,20) = 14.02, *P* < 0.01; IL-6: *F*(1,20) = 15.87, *P* < 0.01; 5 months: IL-1β: *F*(1,20) = 18.08; *P* < 0.01; TNF-α: *F*(1,20) = 12.09, *P* < 0.01; IL-6: *F*(1,20) = 10.71, *P* < 0.01].

**FIGURE 6:**
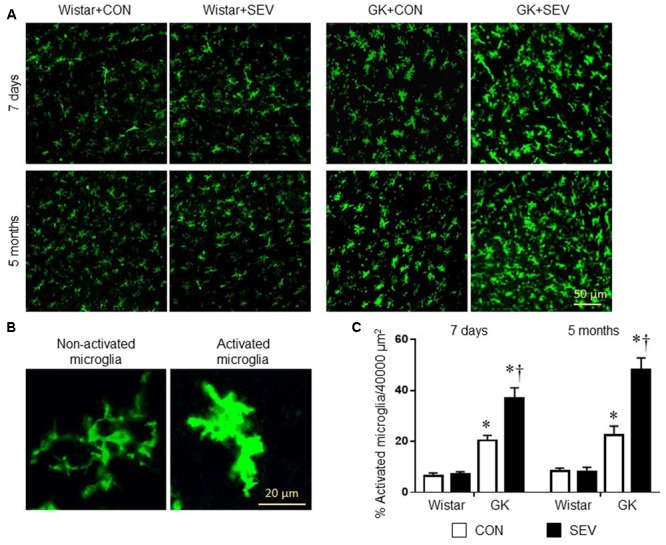
Microglia activation in the hippocampus in type 2 diabetic Goto–Kakizaki (GK) rats and non-diabetic Wistar rats 7 days and 5 months after sevoflurane (SEV) or sham (control, CON) exposure. **(A)** Representative photomicrographs showing CD11b-immunoreactive microglia in the hippocampus in each group. **(B)** Representative photomicrographs showing non-activated and activated microglia in the hippocampus. **(C)** Quantitative analysis of activated microglia in the hippocampus in each group. The number of activated microglia in the hippocampus was higher in GK + CON rats than that in Wistar + CON rats at both time points and was further increased in GK + SEV rats. Data are presented as mean ± SE (*n* = 4 for each group). ^∗^*P* < 0.05 vs. respective Wistar rats at the same time point; ^†^*P* < 0.05 vs. GK + CON rats at the same time point.

Microglia are the primary immune cells in the central nervous system and can be activated by a variety of stimuli, including anesthetics (Rana et al., 2010; Bitzer-Quintero and Gonzalez-Burgos, 2012; Ye et al., 2013). Activated microglia are now recognized to be major sources of proinflammatory cytokines and chemokines within the central nervous system (Bitzer-Quintero and Gonzalez-Burgos, 2012). We therefore examined the effects of the sevoflurane exposure on microglia activation using immunofluorescent study. As shown in **Figure 6**, there are few activated microglia, as defined by strong CD11b immunoreactivity, an enlarged soma, fewer and shorter processes, in the hippocampus in two groups of Wistar rats at 7 days or 5 months after sevoflurane exposure. In contrast, activated microglia were clearly observed in the hippocampus in both groups of GK rats at each time point. Moreover, the number of activated microglia was significantly greater in GK rats receiving sevoflurane exposure when compared with GK control rats [7 days: effect of diabetes: *F*(1,12) = 87.07, *P* < 0.01; effect of SEV: *F*(1,12) = 13.65, *P* < 0.01; 5 months: effect of diabetes: *F*(1,12) = 73.5, *P* < 0.01; effect of SEV: *F*(1,12) = 15.89, *P* < 0.01].

### Sevoflurane Does Not Alter the Metabolic, Hemodynamic, and Physiologic Parameters in Both Wistar and GK Rats

GK control rats exhibited significantly smaller weight gain [*F*(1,28) = 88.35, *P* < 0.01] during the experimental period as compared to Wistar control rats. Plasma glucose concentrations [*F*(1,28) = 204.6, *P* < 0.01] were significantly higher but plasma insulin levels [*F*(1,28) = 7.44, *P* < 0.01] were significantly lower in GK control rats than in Wistar control rats at 9 and 14 months of age (**Table 1**). These data indicate that GK rats develop diabetes with insulin deficiency during the experimental period. Sevoflurane exposure did not affect body weight, plasma glucose concentrations, and insulin levels in either Wistar or GK rats.

**Table 1:** Metabolic parameters.

	Wistar+CON	Wistar+SEV	GK+CON	GK+SEV
**Variables measured at age of 9 months**				
Body weight (g)	436 ± 5	430 ± 8	362 ± 7^∗^	357 ± 5^∗^
Glucose (mg/dL)	113 ± 2	118 ± 3	235 ± 10^∗^	239 ± 8^∗^
Insulin (μg/L)	2.81 ± 0.32	2.79 ± 0.40	1.25 ± 0.28^∗^	1.23 ± 0.36^∗^
**Variables measured at age of 14 months**				
Body weight (g)	472 ± 6	468 ± 10	374 ± 5^∗^	371 ± 9^∗^
Glucose (mg/dL)	125 ± 4	120 ± 7	218 ± 9^∗^	212 ± 6^∗^
Insulin (μg/L)	3.24 ± 0.37	3.30 ± 0.41	0.87 ± 0.10^∗^	0.85 ± 0.11^∗^

*Data are presented as mean ± SE (*n* = 6–10 for each group). ^∗^*P* < 0.05 versus respective Wistar rats at the same time point.*

Before sevoflurane exposure, mean BP [*F*(1,28) = 18.61, *P* < 0.01] was significantly increased in GK control rats than in Wistar control rats, however, heart rate, the acidity (pH), arterial carbon dioxide tension (PaCO_2_), arterial oxygen tension (PaO_2_), and arterial oxygen saturation (SaO_2_) was similar between two control groups (**Table 2**). Notably, sevoflurane exposure did not alter any of these parameters in either Wistar or GK rats. These data excluded the possibility that the molecular and behavioral changes observed in the study were due to hemodynamic or physiologic side effects of sevoflurane.

**Table 2:** Hemodynamic and physiologic parameters before (0 h) and 4 h after sevoflurane exposure.

	MBP (mmHg)	HR (beat/min)	pH	PaCO_2_ (mmHg)	PaO_2_ (mmHg)	SaO_2_ (%)
**0 h**						
Wistar+CON	100 ± 2	329 ± 18	7.41 ± 0.05	38.2 ± 3.1	163 ± 12	98.6 ± 2.2
Wistar+SEV	101 ± 4	332 ± 15	7.40 ± 0.04	40.1 ± 3.5	162 ± 17	98.1 ± 3.9
GK+CON	119 ± 5^∗^	330 ± 11	7.39 ± 0.05	39.7 ± 3.0	166 ± 19	98.5 ± 2.7
GK+SEV	121 ± 4^∗^	335 ± 13	7.40 ± 0.04	40.4 ± 3.3	160 ± 14	98.3 ± 3.0
**4 h**						
Wistar+CON	97 ± 5	320 ± 16	7.39 ± 0.06	38.5 ± 3.9	162 ± 15	98.8 ± 3.3
Wistar+SEV	99 ± 3	328 ± 20	7.38 ± 0.05	39.1 ± 3.7	161 ± 12	98.2 ± 3.4
GK+CON	120 ± 6^∗^	327 ± 14	7.41 ± 0.05	39.2 ± 3.4	163 ± 16	98.6 ± 3.6
GK+SEV	117 ± 5^∗^	325 ± 19	7.39 ± 0.04	40.0 ± 3.5	165 ± 18	98.5 ± 4.2

*MBP, mean blood pressure; HR, heart rate; PaCO_*2*_, arterial carbon dioxide tension; PaO_*2*_, arterial oxygen tension; SaO_*2*_, arterial oxygen saturation. Data are presented as mean ± SE (*n* = 6–10 for each group), ^∗^*P* < 0.05 versus respective Wistar rats at the same time point.*

## Discussion

The major findings of this study are as follows: (1) sevoflurane exposure induces exaggerated and persistent cognitive decline in type 2 diabetic GK rats, whereas it has no effects on cognitive function in Wistar rats; (2) sevoflurane exposure further increases expression of proinflammatory cytokines and activation of microglia in the hippocampus in GK rats but not in Wistar rats. Taken together, these data suggest that type 2 DM increases sensitivity and susceptibility to the insult from sevoflurane exposure, which aggravates neuroinflammation in the hippocampus, contributing to the acute cognitive decline and the persistence of cognitive impairment.

In the behavioral paradigms that we used to interrogate cognitive domains of learning and memory, we found that Wistar control rats and Wistar rats that received sevoflurane did not differ in either TFC 7 days after sevoflurane exposure or in the MWM 5 months after sevoflurane exposure. This finding is consistent with recent reports that a moderate duration of sevoflurane exposure did not impair acquisition learning or memory in both young adult and aged rats (Callaway et al., 2012; Shen et al., 2013). Several experimental studies have shown that sevoflurane induces neurodegenerative symptoms including apoptosis, inflammation, and Aβ accumulation in the hippocampus, resulting in impairment of both short-term and long-term cognitive function (Jevtovic-Todorovic et al., 2003; Tian et al., 2015; Xie et al., 2015), especially in aged animals (Tian et al., 2015). The discrepancy of these results may be ascribed to the differences in animal species and age, the dosage of anesthetic, and/or the time points of the performance of the cognitive tests. Interestingly, our data showed that GK control rats, when compared with Wistar control rats, had less freezing in the TFC test at 9 months of age, longer latency to find the submerged platform as well as reduced number of platform crossings and percent time spent in third quadrant in the MWM test at the end of experiments, indicating impairment in their learning abilities and memory capabilities. These findings are in agreement with previous studies showing that the development of type 2 DM may causes cognitive dysfunction (Moreira et al., 2007; Li et al., 2013b; Zhao et al., 2013), which is associated with an increased risk of developing all forms of dementia, including vascular dementia and Alzheimer’s disease (Biessels et al., 2006; Kopf and Frolich, 2009). Importantly, our data showed that GK rats receiving sevoflurane exhibited both an early exacerbation of memory decline as well as a persistent deterioration in both learning and memory, when compared with GK control rats. Of note, non-cognitive factors that could have contributed to the behavioral assessments, such as altered spontaneous movement in TFC or the swimming speed in MWM, were not significantly different among four experimental groups. Collectively, these findings demonstrate that a moderate duration of sevoflurane exposure has no effect on cognitive function under physiological conditions, but it leads to exacerbation of the acute cognitive decline and the persistence of cognitive impairment under type 2 diabetic conditions. To our knowledge, this is the first study to determine the influence of type 2 DM on anesthetic-induced cognitive impairment.

Although several mechanisms have been suggested to be associated with cognitive dysfunction, accumulating evidence indicates that microglial activation and subsequent neuroinflammation play major roles in the development of cognitive dysfunction. Microglial cells are the resident macrophages in the central nervous system and have been demonstrated to play an important role in the brain’s innate immunity and neuroinflammatory processes (Block, 2014). Although microglia have a beneficial healing effect, activation of microglia is also considered to be toxic to the neighboring neurons via generating cytotoxic mediators including proinflammatory cytokines IL-1β, TNF-α, and IL-6 (Burm et al., 2015; Qiu et al., 2016). Indeed, microglial activation and increased inflammatory cytokines in the brain are deeply involved in the cognitive dysfunction associated with various neurodegenerative disorders including Parkinson’s disease, Alzheimer’s disease, multiple sclerosis, and type 2 DM (Muriach et al., 2014; Srodulski et al., 2014; Lee et al., 2015; Schmole et al., 2015; Qiu et al., 2016; Wu et al., 2016). Moreover, recent studies have demonstrated that surgery or anesthetics can induce microglial activation and neuroinflammation, contributing to cognitive impairment (Wang et al., 2014, 2016; Tian et al., 2015; Qiu et al., 2016). Interventions that reduce microglial activation and neuroinflammation in the brain can attenuate surgery- or anesthetic-induced cognitive impairment (Li et al., 2014; Tian et al., 2015; Qiu et al., 2016; Wang et al., 2016). Our data revealed that there were no differences in expression of proinflammatory cytokines IL-1β, TNF-α, and IL-6 and the number of activated microglia in the hippocampus between Wistar control rats and Wistar rats receiving sevoflurane 7 days or 5 months after sevoflurane exposure. However, compared with Wistar control rats, GK control rats exhibited significant increases in expression of proinflammatory cytokines and the number of activated microglia in the hippocampus at both time points. Moreover, expression of proinflammatory cytokines and the number of activated microglia in the hippocampus were further augmented in GK rats receiving sevoflurane compared with GK control rats. These molecular data were consistent with behavioral findings, suggesting that microglia-mediated neuroinflammation in the hippocampus contributes to cognitive dysfunction in type 2 DM, and that sevoflurane exposure induces exacerbation of the cognitive impairment in type 2 diabetic rats due to aggravation of neuroinflammation in the hippocampus.

The mechanisms by which sevoflurane aggravates neuroinflammation in the hippocampus of type 2 diabetic rats remain unclear. In this study, rats received sevoflurane at 9 months of age which could be considered as middle adulthood. We speculate that neuroinflammation may be prevented by anti-inflammatory counteregulatory mechanisms that maintain normal brain function in non-diabetic animals at this time point. The anti-inflammatory mechanisms in the brain may involve anti-inflammatory external signals (including anti-inflammatory cytokines, transforming growth factor-β, IL-10 and IL-1 receptor antagonist) and intracellular mediators (including endogenous cytoprotective genes or peroxisome proliferator-activated receptors) that may be expressed or activated in the brain in response to proinflammatory stimuli to prevent the inflammatory process and the injury (Tedgui and Mallat, 2001). Under diabetic conditions, these anti-inflammatory external signals or mediators may be impaired or down-regulated, resulting in increased susceptibility to proinflammatory stimuli (including sevoflurane) and subsequent aggravation of neuroinflammation. Notably, sevoflurane-induced aggravation of neuroinflammation in the hippocampus of GK rats was observed even 5 months after sevoflurane exposure. This observation suggests that, under diabetic conditions, sevoflurane exposure might cause permanent changes in some key regulators of proinflammatory gene expression, such as the transcription factor nuclear factor-kappa B. Further studies are needed to elucidate the underlying mechanisms.

Insulin in the brain has been suggested to play an important role in promoting learning and memory function (Zhao and Alkon, 2001). For example, intranasal insulin administration has been demonstrated to improve glucose uptake and age-related cognitive decline in older people and aged animals (Reger et al., 2008; Maimaiti et al., 2016). Additionally, intranasal insulin administration improves memory function, augments synaptic proteins, and significantly reduces microglia-mediated inflammation in the hippocampus in Alzheimer’s disease or traumatic brain injury in animals (Chen et al., 2014; Brabazon et al., 2017). Moreover, a recent study showed that sevoflurane administration caused severe hepatic insulin resistance in a canine model (Kim et al., 2016). Insulin resistance induced by sevoflurane might also happen in the brain. In the present study, GK control rats showed significantly lower levels of plasma insulin when compared with Wistar control rats. Thus, we could not exclude the possibility that the cognitive decline observed in GK rats or exaggeration of cognitive dysfunction induced by sevoflurane were partly due to reduced insulin levels or increased insulin resistance in the brain.

With the increasing prevalence of type 2 diabetic patients undergoing surgery, and the increased risk of complications associated with type 2 DM, the appropriate perioperative assessment and management are necessary. The present study demonstrates that anesthetic sevoflurane induces exaggerated and persistent cognitive decline in a type 2 diabetic rat model probably by aggregating hippocampal inflammation. The findings from this study may provide useful information regarding clinical anesthetic choice for type 2 diabetic patients in the future.

## Author Contributions

DL and LZ conceived and designed the experiments. DL, LLiu, LLi, XL, BH, CZ, ZZ, CW, PD, XZ, and BY performed the experiments. DL, LLiu, and LZ analyzed the data. DL and LZ wrote the paper.

## Conflict of Interest Statement

The authors declare that the research was conducted in the absence of any commercial or financial relationships that could be construed as a potential conflict of interest.
